# Irigenin, a novel lead from *Iris confusa* for management of *Helicobacter pylori* infection with selective COX-2 and *Hp*IMPDH inhibitory potential

**DOI:** 10.1038/s41598-022-15361-w

**Published:** 2022-07-06

**Authors:** Passent M. Abdel-Baki, Moshera M. El-Sherei, Amal E. Khaleel, Marwa M. Abdel-Aziz, Mona M. Okba

**Affiliations:** 1 Department of Pharmacognosy, Faculty of Pharmacy, Cairo University, Cairo, 11562 Egypt; 2 Regional Center for Mycology and Biotechnology (RCMB), Al-Azhar University, Cairo, 11651 Egypt

**Keywords:** Target validation, Peptic ulcers

## Abstract

The development of new natural drugs for *Helicobacter pylori* (*H. pylori*) management has recently received significant attention. *Iris confusa* (*I. confusa*) was long used for the treatment of bacterial infections and gastritis. This study aimed at evaluating its effect on management of *H. pylori* infection and exploring its bioactive metabolites. The inhibitory potential of the polar (PF), non-polar (NPF) fractions and the isolated compounds against *H. pylori* using 3-(4,5-dimethylthiazol-2-yl)-2,5-diphenyl-2H-tetrazolium bromide (MTT) assay in addition to their cyclooxygenases (COX-1 and COX-2), and nitric oxide (NO) inhibitory activities were assessed. The most biologically active compound was tested for its selective *H. pylori* inosine-5′-monophosphate dehydrogenase (*Hp*IMPDH) inhibitory potential. Chromatographic purification of PF and NPF allowed isolation of tectoridin, orientin, irigenin, tectorigenin, isoarborinol and stigmasterol. The PF exhibited significant anti-*H. pylori* (MIC 62.50 µg/mL)*,* COX-1, COX-2 (IC_50_ of 112.08 ± 0.60 and 47.90 ± 1.50 µg/mL respectively, selectivity index SI of 2.34), and NO (IC_50_ 47.80 ± 0.89 µg/mL) inhibitory activities, while irigenin was the most potent isolated compound. Irigenin was found to have a promising activity against *Hp*IMPDH enzyme (IC_50_ of 2.07 ± 1.90 μM) with low activity against human *h*IMPDH2 (IC_50_ > 10 μM) than clarithromycin, assuring its selectivity. Overall, *I. confusa* and its isolated compounds may serve as a potential source of plant-based drugs for *H. pylori* control. This study scientifically validated the claimed anti-bacterial activity of *I. confusa* and revealed irigenin potential as a novel lead exhibiting anti *H. pylori* activity in a first record.

## Introduction

*Helicobacter pylori* (*H. pylori*) is a Gram negative bacterium that colonizes 50% of the world population stomach^1^. It is considered as a main factor of chronic gastritis and gastric/duodenal ulcers^2^. A marked reduction in gastric or duodenal ulcers recurrence rates associated with *H. pylori* infection was achieved by curing the infection^3^. *H. pylori* infection is the major risk factor associated with gastric cancer development^4^. *H. pylori* infection triggers the transition from normal mucosa to non-atrophic gastritis and initiates precancerous lesions which after that progress to intestinal metaplasia and atrophic gastritis^5^. *H. pylori* is classified by the International Agency for Research on Cancer^6^ and World Health Organization (WHO) as group one carcinogen.

Noticeable infiltration of inflammatory cells along with release of the inflammatory mediators occur due to *H. pylori* colonization in the gastric mucosa^7^. Also, *H. pylori* infection activates the mucosal defense mechanisms^2^. Prostaglandins (PGs) play an important role as mucosal defense factors protecting the gastric mucosa against injury by inducing gastric mucus and bicarbonate secretion, inhibiting acid secretion and decreasing gastric motility^8^. PGs are synthesized through cyclooxygenase (COX) enzymes.

COX-1 is constitutively expressed in the stomach, while COX-2 expression is induced at inflammation sites^9^. COX-1 participates in PGs production under normal physiological conditions. Thus, it is regarded as the isoform generating PGs associated with housekeeping functions, as protective action on the gastric mucosa against ulceration. On the other hand, COX-2 is involved in the PGs production associated with the development of several diseases^10^ such as autoimmune diseases including rheumatoid arthritis^11,12^, allergic sign and symptoms^13^, Alzheimer’s disease^14^, and the development and prognoses of numerous cancers^15^. Moreover, COX-2 was proved to be involved in gastrointestinal cancer^16^. *H. pylori* increases COX-2 expression consequently stimulating the release of PGs in *H. Pylori* associated premalignant and malignant gastric lesions^17^.

Thus, selective COX-2 inhibitors decrease PGs levels in inflammatory sites only and they have no effect on gastric mucosal PGs levels or integrity . On the other hand, specific COX-2 inhibitors, which cause no significant inhibition of COX-1, have been reported to delay healing of ulcers^18,19^.

Inflammation predisposes to the development of cancer and promotes all stages of tumorigenesis. inflammation drives tumor initiation, growth, progression, and metastasis^20^. Therefore, NSAIDs can aid in cancer prevention^21^ and a dual anti-*H. pylori*/anti-inflammatory compound could both help in eradicating the microorganism and avoid the development of *H. pylori*-related cancers.

Safe NSAID COX-2-inhibitors must have selectivity indices (IC_50_ COX-2/IC_50_ COX-1) ranging from 5 to 50^22^. Since super selective COX-2-inhibitors like Rofecoxib were withdrawn from the market because of the increased risk of heart attack and stroke. Thus, it becomes essential to find alternative safe NSAID COX-2-inhibitors. Most of the natural products tend to be more selective towards COX-1 than COX-2 but they can be further modified to increase COX-2 selectivity^23^.

Antibiotics such as amoxicillin, tetracycline, metronidazole or clarithromycin in addition to proton pump inhibitors or bismuth salts are considered as the only known current therapy for *H. pylori* infection^24^. This triple therapy is not always successful in eradication of the infection. Reduced treatment efficacy can occur due to emergence of antibiotic resistant *H. pylori*. Moreover, non-steroidal anti-inflammatory drugs (NSAIDs), which have been commonly used for COX-2 inhibition, resulted in serious adverse effects as gastrointestinal bleeding and gastric mucosa damage after prolonged intake^25^. *H. pylori* infection and NSAIDs are the main causative risk factors for gastroduodenal ulcers^26^. Therefore, discovering alternative therapy for management of *H. pylori* infection and the associated inflammation is crucial to overcome the undesirable effects of the currently used therapy.

Since ancient times, the use of medicinal plants has been adopted and considered the origin of modern medicine^27^. Many plants are not deeply investigated, although they have been reported to contain various secondary metabolite classes of well known anti-microbial and anti-inflammatory potentials.

*Iris* is the largest genus in the family Iridaceae with the greatest taxonomic diversity^28^. Numerous interesting secondary metabolites such as triterpenoids and phenolics; flavonoids and xanthones were detected in genus *Iris*. Extracts and pure compounds of the irises were reported to have several biological activities such as antimicrobial, anti-oxidant, anti-inflammatory, phytoestrogenic, anti-diabetic, anti-tumor, and anti-cholinesterase activities^29,30^.

Long time ago irises were used for the treatment of bacterial infections in the Mongolian folk medicine^31^. Irises have been found in Karnak temple engraved on a marble panel of the ancient Egyptians^32^. In traditional Chinese medicine, the rhizomes of *Iris confusa*, one of the irises native to western China, were used extensively to treat tonsillitis, acute bronchitis, colitis, and chronic atrophic gastritis^33^.

Reviewing the current literature, very few reports were traced concerning *I. confusa* Sealy (bamboo iris) phytochemical constituents^29,34^. In addition, nothing was traced about the biological activity of its isolated compounds except the study conducted by Chen et al. 2018 who studied their anti- hepatitis B potential^34^. In a continuation of our interest in exploring irises metabolites and their possible activities^35^, *I. confusa* was selected for this study. Its polar and non-polar fractions were subjected to chromatographic fractionation and purification with the aim of isolation of its major constituents and testing their anti *H. pylori* and selective anti-inflammatory potencies.

## Material and methods

### Plant material, extraction and fractionation

*Iris confusa* Sealy was collected from Al-Mansouria, Giza, Egypt in the flowering stage after permission from Agricultural Research Center, Giza, Egypt in compliance with the national guidelines. The plant material identity was kindly authenticated and verified by Dr Nina Davies, the curator of the African Iridaceae, Royal Botanic Gardens, Kew, London, UK. Specimen was deposited in Pharmacognosy Department Herbarium, Cairo University (registration no. 15.1. 2019I). The air-dried powdered underground parts of *I. confusa* were extracted and fractionated according to Salem et al*.* yielding polar fraction (PF) and non-polar fraction (NPF)^36^. Collection of plant material, complied with the institutional, national, and international guidelines and legislation.

### Major constituents isolation

The PF and NPF were screened on pre-coated silica gel 60 F_254_ using solvent system S_1_ (methylene chloride:methanol:formic acid 85:15:0.2 *v/v/v*) and S_2_ (methylene chloride:methanol 97:3 *v/v*), respectively. The spots were examined in UV light before and after ammonia vapour exposure and AlCl_3_ spraying and after being sprayed with *p*-anisaldehyde/H_2_SO_4_ followed by heating at 110 °C. PF and NPF purification using a vacuum liquid chromatography column (VLC) packed with silica gel H 60, ion exchange resin (diaion HP-20), sephadex LH 20 and silica gel 60 were illustrated in detail in Fig. S1.

Similar fractions were pooled together and evaporated to dryness under reduced pressure yielding compounds P_1_ (yellow powder, R_*f*_ = 0.28, S_1_), P_2_ (yellow powder, R_*f*_ = 0.18, S_1_), P_3_ (yellow crystals, R_*f*_ = 0.69, S_1_) and P_4_ (yellow crystals, R_*f*_ = 0.55, S_1_) from the PF and N_1_ (white crystals, R_*f*_ = 0.62, S_2_) and N_2_ (white crystals, R_*f*_ = 0.43, S_2_) from the NPF.

### Determination of anti-*Helicobacter pylori* activity

The anti-*Helicobacter pylori* activity of the PF and NPF as well as the isolated compounds was evaluated against *H. pylori* ATCC 700392 (type strain, obtained from the American Type Culture Collection unit (ATCC), using microwell dilution method and clarithromycin as standard drug^37^. The detailed procedures were described in the supplementary file.

### Determination of COX-1 and COX-2 inhibitory activity

The tested samples and the standard drugs (ibuprofen and celecoxib) were prepared in dimethyl sulfoxide and subsequent eight twofold dilutions (125–0.98 µg) were carried out in a 96-well plate. The inhibitory COX activity was assayed colourimetrically by examining the inhibition of the ovine COX-1 and human recombinant COX-2 enzymes as described by George et al.^38^, using a COX inhibitor screening assay kit. The detailed procedures were described in the supplementary file.

### Determination of nitric oxide (NO) inhibitory activity

The macrophage cell line, RAW 264.7 was obtained from Vaccines, Sera and Drugs Egyptian Company (VACSERA). It was cultured in (RPMI, 1640) medium supplemented with 10% fetal bovine serum and 1% gentamicin^39^. The detailed procedures were described in the supplementary file.

### In vitro* Hp*IMPDH inhibition assay

The most potent isolated compound, as revealed from the previous assays, was screened at different concentrations (10–0.078 µM) in triplicates. The assay was carried out according to Galal^40^ and was described in detail in the supplementary file.

### In vitro* hIMPDH2* inhibition assay

Human inosine-5′-monophosphate dehydrogenase (*h*IMPDH2) was purchased from NovoCIB SAS (Lyon, France). It was used for the in vitro screening of the most potent isolated compound, as revealed from the previous assays, against *H. pylori* and the standard drug at the concentrations (10–0.078 µM) in triplicates^40^. The detailed procedures were described in the supplementary file.

### Quantitative determination of the main phytochemical classes and antioxidant assay

#### Total phenolic content (TPC)

Determination of TPC was carried out according to the European Pharmacopeia procedure^41^, using the Folin–Ciocalteu colourimetric method. It depends on measuring the intensity of the blue colour produced in correlation to the reducing power of existing phenolics. Determinations were carried out in triplicates; results are the mean values ± standard deviations and expressed as μg gallic acid equivalent (GAE) per mg dry fraction (μg GAE/mg).

#### Total flavonoid content (TFC)

TFC was determined based on measuring the intensity of the yellow colour developed upon reaction of flavonoids with aluminium chloride reagent^42^. Quercetin was used to compute the standard calibration curve. Determinations were carried out in triplicates; results were the mean values ± standard deviation and expressed as μg quercetin equivalent (QE) per mg dry fraction (μg QE/mg).

#### Total triterpene content (TTC)

TTC was determined based on measuring the intensity of the red–purple colour developed upon reaction of perchloric acid-oxidized triterpenes in glacial acetic acid with vanillin^43^. Ursolic acid was used to compute the standard calibration curve. Determinations were carried out in triplicates; results were the mean values ± standard deviations and expressed as μg ursolic acid equivalent (UAE) per mg dry fraction (μg UAE/mg).

#### DPPH assay

The DPPH anti-oxidant assay was carried out as described by Romano et al.^44^ with some modifications. The PF and NPF of *I. confusa* underground parts were separately dissolved in methanol by the aid of sonication to give a set of serial dilutions for each sample. Experiments were performed in triplicates using a 96-wells plate. The reaction mixture in each case consisted of 22 μL of the tested sample and 200 μL of 0.004% DPPH in methanol. The non-quenched DPPH radicals were assessed spectrophotometrically at λmax = 492 nm using a micro-plate reader.

### Ethical statement

Collection of the plant material, complied with relevant institutional, national, and international guidelines and legislation.

## Results

### Purification of PF and NPF

Chromatographic purification of *I. confusa* underground parts PF allowed the isolation of three isoflavonoids; tectoridin (P_1_), irigenin (P_3_), and tectorigenin (P_4_), and one flavone, orientin (P_2_). On the other hand, purification of the NPF led to the isolation of one triterpene, isoarborinol (N_1_), and one sterol, stigmasterol (N_2_).

**Figure 1:**
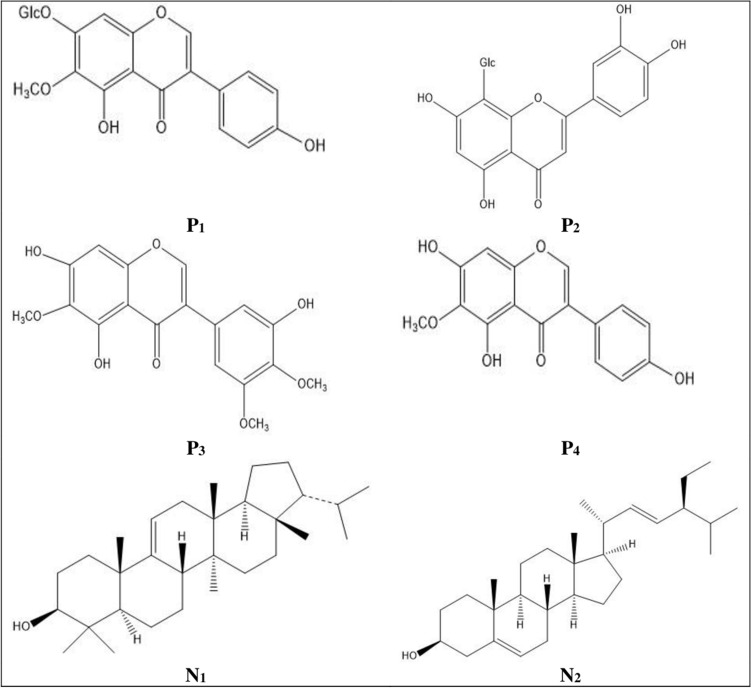
Structure of the isolated compounds. *P*_*1*_ tectoridin, *P*_*2*_ orientin, *P*_*3*_ irigenin, *P*_*4*_ tectorigenin, *N*_*1*_ isoarborinol, *N*_*2*_ stigmasterol.

The isolated compounds (P_1_–P_4_ and N_1_–N_2_) were identified through their physicochemical characters, spectroscopic analysis, UV spectral data and via comparing their ^1^H and ^13^C NMR data with the published data (Tables S1–S4 in the supplementary file). Their structures are shown in Fig. 1.

### *Helicobacter pylori* inhibitory activity

**Figure 2:**
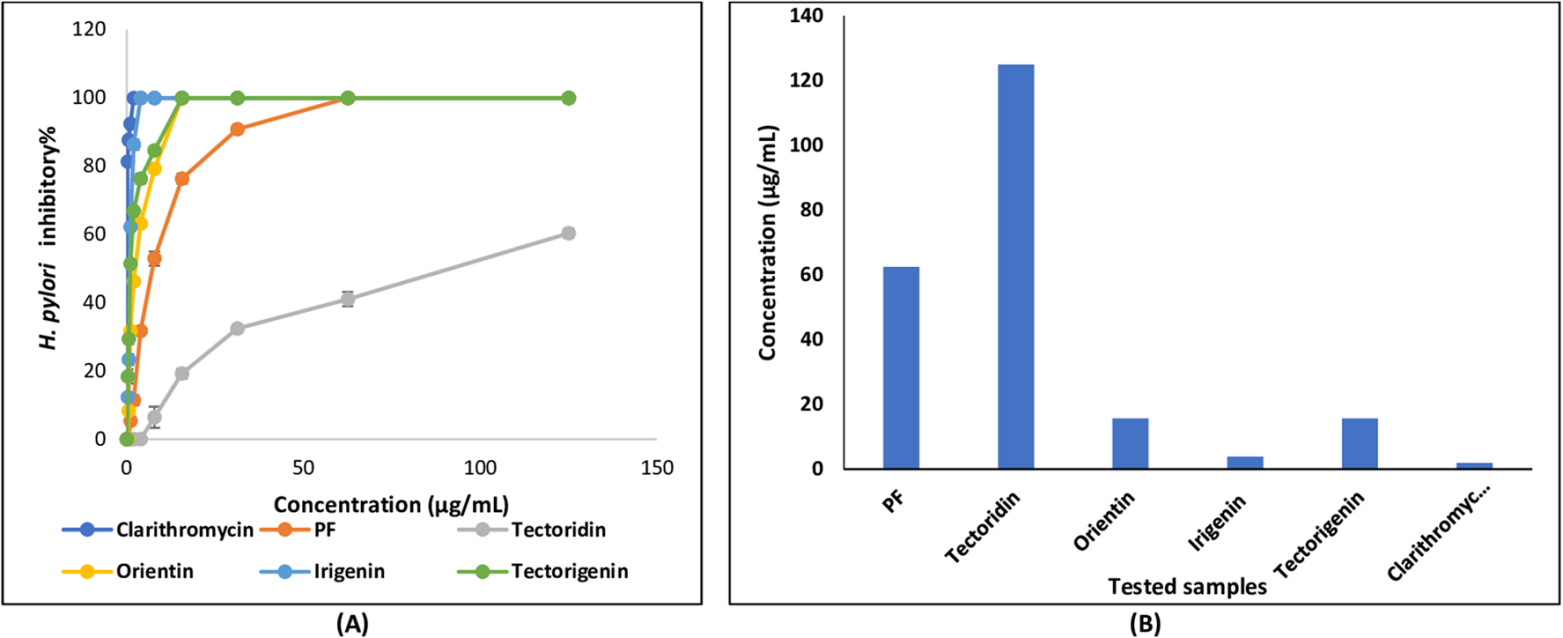
(**A**) Percentage inhibition and (**B**) MIC values of *I. confusa* underground PF and isolated compounds against *H. pylori*. All determinations were carried out in a triplicate manner and values are expressed as the mean ± SD; *PF* polar fraction.

*I. confusa* underground parts PF inhibited the growth of *H. pylori* with MIC value of 62.50 μg/mL (Table 1 and Fig. 2). Compounds isolated from PF showed more potent activity than their crude fraction (irigenin: MIC 3.90 μg/mL; orientin: MIC 15.53 μg/mL and tectorigenin: MIC 15.63 μg/mL) except tectoridin which showed MIC > 125 μg/mL. The most potent isolated compound was irigenin (MIC 10.82 μM) followed by orientin (MIC 34.64 μM) and tectorigenin (MIC 52.05 μM). *I. confusa* underground parts NPF as well as its isolated compounds (isoarborinol and stigmasterol) exhibited no *H. pylori* inhibitory potential.

**Table 1:** Minimum inhibitory concentration of *I. confusa* underground parts PF, NPF and isolated compounds against *Helicobacter pylori* and their IC_50_ on COX-1, COX-2, and nitric oxide (NO).

	*I. confusa*		Compounds isolated from PF				Compounds isolated from NPF		Positive control*
	PF	NPF	Tectoridin (P_1_)	Orientin (P_2_)	Irigenin (P_3_)	Tectorigenin (P_4_)	Isoarborinol (N_1_)	Stigmasterol (N_2_)	
MIC (*H. pylori*)	62.50^a^	ND	> 125^a^	15.53^a^	3.90^a^	15.63^a^	ND	ND	1.95^a^
			> 270.33^b^	34.64^b^	10.82^b^	52.05^b^	ND	ND	2.61^b^
IC_50_ (COX-1)	112.08 ± 0.60^a^	ND	ND	73.30^a^ ± 0.60	12.70^a^ ± 1.40	122.30^a^ ± 0.90	31.30^a^ ± 1.00	33.60^a^ ± 1.10	8.07^a^ ± 1.40
				163.48^a^ ± 0.60	35.25^b^ ± 1.40	407.31^b^ ± 0.90	73.35^b^ ± 1.00	81.42^b^ ± 1.10	39.12^b^ ± 1.40
IC_50_ (COX-2)	47.90 ± 1.50^a^	> 125^a^	ND	46.50^a^ ± 1.70	3.90^a^ ± 0.86	56.70^a^ ± 1.50	22.10^a^ ± 2.10	25.20^a^ ± 2.10	0.28^a^ ± 0.10
				103.70^b^ ± 1.70	10.83^b^ ± 0.86	188.84^b^ ± 1.50	51.79^b^ ± 2.10	61.06^b^ ± 2.10	0.73^b^ ± 0.10
SI (COX-1/COX-2)	2.34	ND	ND	1.58	3.26	2.16	1.42	1.33	
IC_50_ (NO)	47.80 ± 0.89^a^	ND	ND	55.60^a^ ± 0.86	15.30^a^ ± 1.80	> 125^a^	18.90^a^ ± 1.70	53.80^a^ ± 1.30	2.80^a^ ± 0.69
				124^b^ ± 0.86	42.46^b^ ± 1.80	> 416.31^b^	44.29^b^ ± 1.70	130.36^b^ ± 1.30	12.62^b^ ± 0.69

^a^µg/mL; ^b^µM; *IC*_*50*_ concentration at which the tested sample produces 50% inhibition, *MIC* minimum inhibitory concentration, *ND* not detected, *NPF* non-polar fraction, *PF* polar fraction, *SI* selectivity index; *Positive control clarithromycin for MIC, and L-*N*^6^-(1-iminoethyl) lysine hydrochloride for nitric oxide.

### COX-1 and COX-2 inhibitory activity

**Figure 3:**
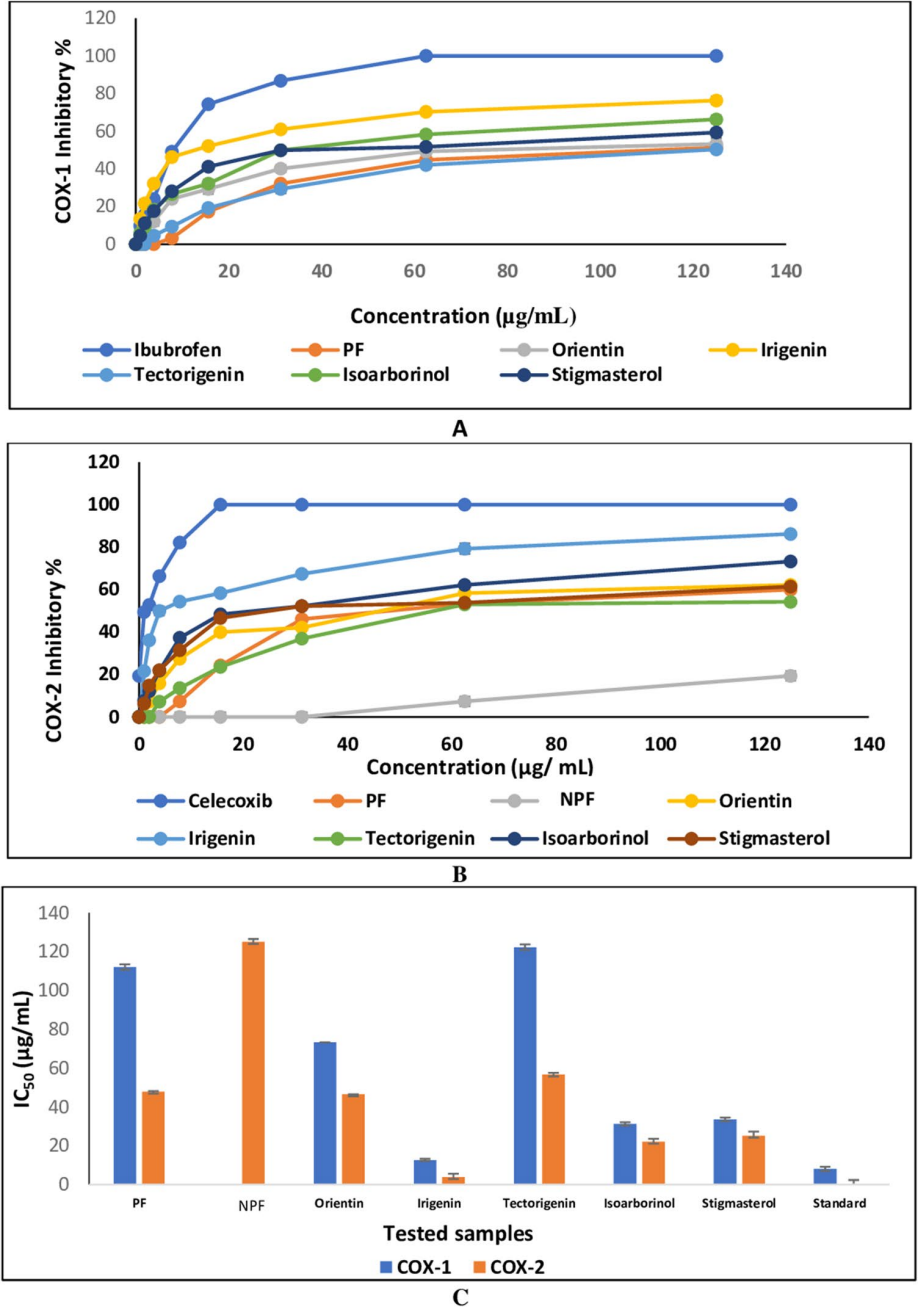
Effect of *I. confusa* underground parts PF, NPF and isolated compounds on (**A**) COX-1 and (**B**) COX-2 inhibition, (**C**) IC_50_ compared to reference drugs. All determinations were carried out in triplicate and values are expressed as the mean ± SD; *NPF* non-polar fraction, *PF* polar fraction, *IC*_*50*_ concentration at which the tested sample produces 50% inhibition, *Standard* ibuprofen for COX-1, celecoxib for COX-2.

*I. confusa* underground parts PF inhibited COX-1 and COX-2 by an IC_50_ of 112.08 ± 0.60 and 47.9 ± 1.50 µg/mL, respectively (Table 1 and Fig. 3). On the other hand, the NPF did not show any inhibition to COX-1 and IC_50_ > 125 µg/mL against COX-2.

The isolated compounds were more potent than the corresponding crude fractions except for tectorigenin (COX-1 IC_50_ of 122.30 ± 0.90 and COX-2 IC_50_ of 56.70 ± 1.50 µg/mL) and its glycoside tectoridin which showed no inhibition against both COX-1 and COX-2. Among the isolated compounds, irigenin was the most potent COX-1 (IC_50_ of 35.25 ± 1.40 μM) and COX-2 (IC_50_ of 10.83 ± 0.86 μM) inhibitor followed by isoarborinol (COX-1 IC_50_ of 73.35 ± 1.00 μM and COX-2 IC_50_ of 51.79 ± 2.10 μM).

The isolated compounds showed higher inhibitory potential against COX-2 than COX-1 enzyme. Among all tested samples, irigenin showed the highest selectivity for COX-2 (SI = 3.26).

### Nitric oxide assay

**Figure 4:**
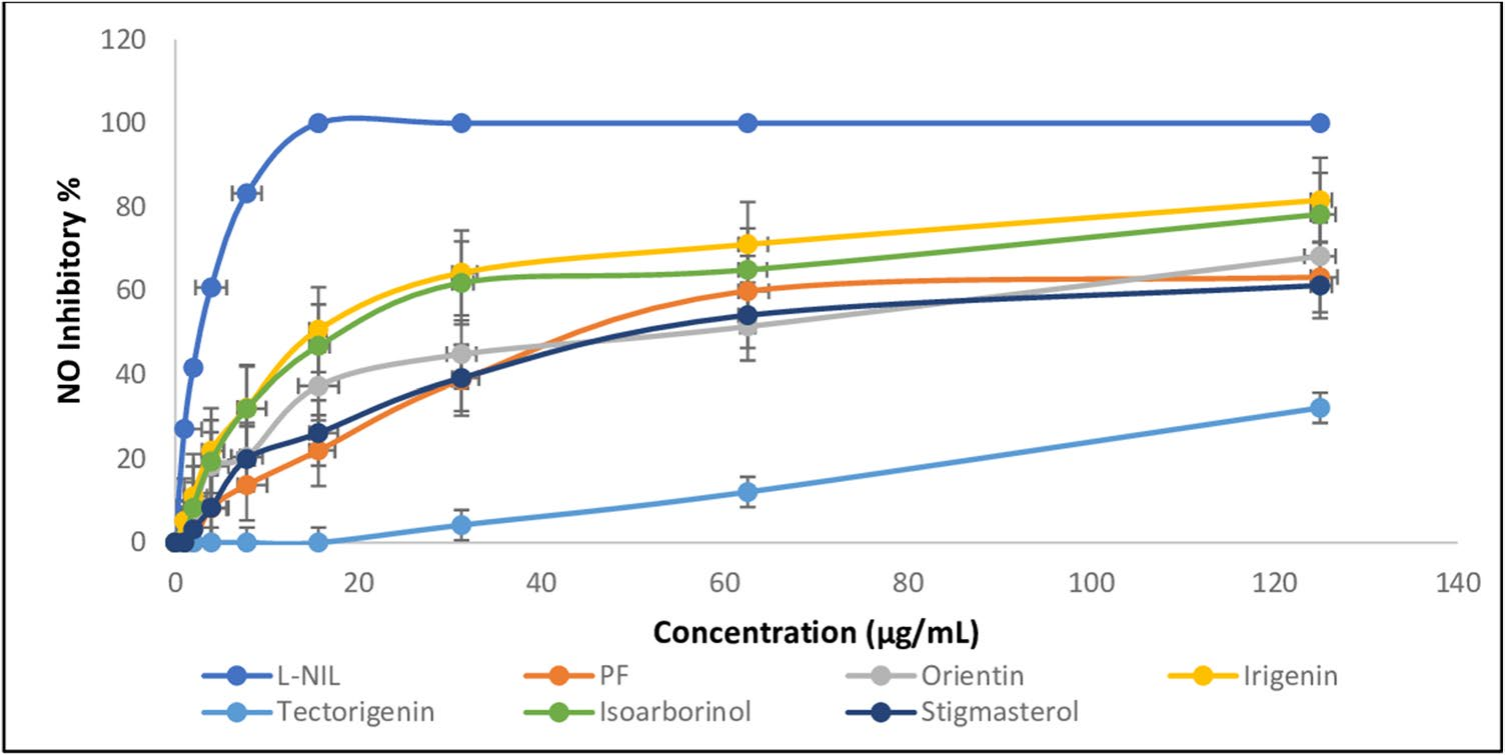
Effect of *I. confusa* fractions and isolated compounds on NO inhibition. All determinations were carried out in triplicate and values are expressed as the mean ± SD; *L*-*NIL* L-*N*^6^-(1-iminoethyl) lysine hydrochloride, *PF* polar fraction.

In the same manner, *I. confusa* underground parts PF exhibited nitric oxide inhibition by an IC_50_ of 47.80 ± 0.89 µg/mL while the NPF was inactive (Table 1 and Fig. 4). Irigenin was the most active tested compound. It showed nitric oxide inhibition by an IC_50_ of 42.46 ± 1.80 μM followed by isoarborinol (IC_50_ of 44.29 ± 1.70 μM).

### IMPDH inhibition assays

On the basis of the previous in vitro assays on *I. confusa* underground parts PF and NPF as well as their isolated compounds (Table 1), the most active compound was irigenin. Thus, irigenin was selected to test its *Hp*IMPDH inhibitory activity by monitoring NADH production at concentration range of 10–0.078 µM compared to clarithromycin as standard drug. Also, to examine irigenin selectivity toward the bacterial IMPDH, human *h*IMPDH2 inhibition potential was assayed.

Results showed that irigenin (P_3_) exhibited potent inhibitory potential against *Hp*IMPDH enzyme with IC_50_ of 2.07 ± 1.90 μM (Table 2). Whereas the IC_50_ value of clarithromycin (standard drug) was 0.51 ± 0.58 μM. In addition, irigenin was less active and safer against *h*IMPDH2 with IC_50_ > 10 μM, compared with clarithromycin (IC_50_ 3.10 ± 2.10 μM), which make sure that its selectivity is toward *Hp*IMPDH in contrast of clarithromycin. *I. confusa* underground parts PF exhibited high DPPH scavenging activity with EC50 of 41.68 ± 6.67 μg/mL. The polar fraction TPC amounted 99.05 ± 0.02 μg GAE/mg dried fraction and TFC amounted 98.31 ± 0.04 μg QE/mg dried fraction while, the non-polar fraction TTC amounted 126.30 ± 0.04 μg UAE/mg dried (Table 3).

**﻿Table 2:** *Hp*IMPDH and *h*IMPDH2 inhibitory activity of irigenin (P_3_) and the standard drug clarithromycin.

Sample conc.	Irigenin (P3)		Clarithromycin	
	*Hp*IMPDH	*h*IMPDH2	*Hp*IMPDH	*h*IMPDH2
10	83.25 ± 0.58	22.68 ± 1.00	100 ± 0.00	76.32 ± 0.50
5	71.08 ± 1.30	10.36 ± 0.72	100 ± 0.00	60.24 ± 1.50
2.5	59.31 ± 1.40	4.81 ± 0.91	81.22 ± 1.60	46.32 ± 2.20
1.25	24.19 ± 1.70	0.00	69.31 ± 2.10	27.32 ± 1.70
0.625	10.25 ± 2.80	0.00	56.06 ± 0.58	19.35 ± 1.50
0.313	6.14 ± 1.90	0.00	38.25 ± 1.50	9.74 ± 0.67
0.156	0.00	0.00	21.82 ± 2.10	0.00
0.078	0.00	0.00	9.14 ± 1.70	0.00
0	0.00	0.00	0.00	0.00
IC_50_ (µM)	2.07 ± 1.90	> 10	0.51 ± 0.58	3.10 ± 2.10

**Table 3:** Total phenolic, flavonoid, and triterpene content of the polar and non-polar fractions of *I. confusa* underground parts and their DPPH scavenging activity.

	TPC μg GAE/mg dried fraction ± SD	TFC μg QE/mg dried fraction ± SD	TTC μg UAE/mg dried fraction ± SD	EC_50_ ± SD (μg/mL)
PF	99.05 ± 0.02	98.31 ± 0.04	25.38 ± 0.04	41.68 ± 6.67
NPF	6.55 ± 0.01	13.03 ± 0.01	126.30 ± 0.04	430.61 ± 2.78

*Average of three determinations; *GAE* gallic acid equivalent, *NPF* non-polar fraction, *PF* polar fraction, *SD* standard deviation, *TPC* total phenolic content, *QE* quercetin equivalent, *TFC* total flavonoid content, *TTC* total triterpene content, *UAE* ursolic acid equivalent. EC_50_ = effective concentration of the sample required to scavenge 50% of the DPPH free radical.

## Discussion

The eradication of *H. pylori* infection has been proven to prevent gastric or duodenal relapses. Direct correlation was observed between the development of gastric adenocarcinoma and the long-term infection with *H. Pylori*. Therefore, antibiotics such as clarithromycin have been used for treatment. The declining eradication rates provoked the search for alternative therapeutic options which should be more effective and safer.

Exploring medicinal plants along with their phytoconstituents for *H. pylori* infection control is not just a way to discover safer pharmaceutical alternatives, but also is a trial to discover a natural affordable effective drug especially in developing countries.

The selection of *Iris* in this study was based on our previous experience with this genus diverse metabolites content and antibacterial potential^29,35^. As well as, *I. confusa* has been used a long time ago in the treatment of bacterial infections and gastritis in traditional Chinese medicine^33^. Hence, the in vitro* H. pylori*, COX-1, COX-2, and NO inhibition potency of *I. confusa* underground parts PF, NPF, and their isolated compounds were assessed. Additionally, the selective IMPDH inhibition activity of the most potent isolated compound, irigenin, was examined.

Purification of the PF led to the isolation of four metabolites (P_1_–P_4_) while two compounds (N_1_–N_2_) were purified from the NPF. Compounds P_1_–P_4_ spectral data were in agreement with the reported data of tectoridin^45^, orientin^46^, irigenin^47^, and tectorigenin^45^. Compounds N_1_–N_2_ spectral data were in agreement with the reported data of isoarborinol^48^ and stigmasterol^49^. This is the first report for the presence of isoarborinol (N_1_) in genus *Iris.* P_1_–P_4_ and N_2_ are herein isolated from *I. confusa* for the first time.

*I. confusa* underground parts PF and its isolated compounds showed significant inhibitory effects on *H. pylori*. Irigenin was superior over tectorigenin as anti-*H. pylori*. This was attributed to the presence of methoxy group at C-4′ in irigenin, which increases the *H. pylori* inhibitory effect than the hydroxyl group in C-4′ of tectorigenin as previously reported by Park et al.^50^.

Although tectorigenin (P4) exhibited certain inhibitory activity against *H. pylori*, its corresponding *O*-glycoside; tectoridine (P1) exhibited no activity. This was attributed to the effect of glycosylation at 7-OH of tectoridin which caused dramatic decrease in the *H. pylori* inhibitory activity compared to its aglycone tectorigenin. This dramatic decrease in the activity of the isoflavone glycosides was well documented before^50^. The observed anti-*H. pylori* activity of orientin matched that previously reported by Król-Kogus et al.^51^.

The inflammation usually associated with *H. pylori* infection drained the authors interest to explore the COX-1 and COX-2 inhibitory potential. The need to explore drugs with selective COX-2 inhibitory potential that are able to decrease PGs dependent inflammation while maintaining protective gastric mucosal PGs synthesis intact has increased in recent decades. Finding COX-2 inhibitors, but not specific and still having a degree for COX-1 inhibition^18,52^ was the main target.

Irigenin was found to exhibit COX-2 selective inhibitory activity three times more than COX-1 (SI = 3.26) followed by tectorigenin (SI of 2.16) and orientin (SI of 1.58).

Nitric oxide is free oxygen radical and has cytotoxic effect in pathological processes, particularly in inflammatory disorders. Nitric oxide is a potent proinflammatory molecule secreted during inflammation and causes vasodilation and cellular migration. At higher concentrations, it downregulates adhesion molecules and induces apoptosis of inflammatory cells. Inhibition of NOS (inducible nitric oxide synthase) is beneficial for the treatment of inflammatory disease^53–56^. Irigenin exhibited significant potential in inhibiting nitric oxide followed by isoarborinol.

Many microbial infections are characterized by rapid proliferation that is supported by guanine nucleotide pool expansion in the rapidly dividing cells. Inosine 5ʹ‐monophosphate dehydrogenase (IMPDH), an important enzyme required for the new synthesis of guanine nucleotides, is an interesting target for antimicrobial drug development^57^. This enzyme, IMPDH, catalyzes the oxidation of inosine 5ʹ‐monophosphate (IMP) to xanthosine 5ʹ‐monophosphate (XMP) leading to reduction of nicotinamide adenine dinucleotide (NAD^+^), which is an important step in guanine nucleotides de novo synthesis. IMPDH inhibition surely leads to major fall in guanine nucleotide pool which consequently blocks proliferation^57^.

The most active anti *H. pylori* and anti-inflammatory tested compound; irigenin (P_3_) was chosen to test its inhibitory potential against the bacterial *Hp*IMPDH enzyme and to evaluate its selectivity relative to the host enzyme (hIMPDH2). It was found to have promising activity against the *Hp*IMPDH enzyme, and to be safer and less active against the *h*IMPDH2, which reflected its selectivity.

Therefore, *I. confusa* underground parts and its isolated compounds can be used as excellent therapy to control gastric ulcers via eradication of *H. pylori* and exerting its anti-inflammatory potentials.

The PF of *I. confusa* underground parts exhibited high DPPH scavenging activity which was in accordance with its high TPC and TFC. The DPPH scavenging activity could be attributed to its total phenolic and flavonoid contents^58^. The observed anti-oxidant potential of the NPF could be attributed to the recently detected *I. confusa* xanthones and triterpenoids^29^. Xanthones and triterpenoids metabolites are well documented anti-oxidants^59,60^.

In recent years, irigenin, isolated from *Belamcanda chinensis* (Iridaceae) has been a hot research topic due to its important bioactivities. Irigenin controlled the metastatic progression in lung carcinoma cells^61^. It was recently proved to exhibit beneficial potentials in management of cardiac injuries. It alleviated doxorubicin (DOX)-induced cardiotoxicity^62^ and protected HUVECs (human umbilical vein endothelial cell lines) from angiotensin II-induced oxidative stress and apoptosis injury^63^. Irigenin exhibited inhibitory effects on prostaglandin E_2_ and nitric oxide production in murine macrophage RAW 264.7 cells^64^.

Our results presented irigenin (P_3_) as a new anti‐*H. pylori*, COX, NO and *Hp*IMPDH inhibitor beside being previously reported to significantly enhance TRAIL-regulated apoptosis in Tumor necrosis factor-related apoptosis-inducing ligand (TRAIL) resistant gastric cancer cells^65^. This highlights the promising expected results upon future testing of its in vivo potential in gastric ailments.

It is noteworthy to mention that, this is the first time to isolate irigenin from *I. confusa* by such classical chromatographic techniques. Thus, we herein present *I. confusa* as another natural source of irigenin other than *Belamcanda chinensis* (Iridaceae).

Besides irigenin, isolated in this study, the anti-*H. pylori* activity of *I. confusa* underground parts is attributed to its content of flavonoids, isoflavonoids, and xanthones which were recently reported in the PF^29^. Flavonoids and isoflavonoids react with superoxide anion, lipid peroxy, and hydroxyl radicals thus can protect the gastric mucosa against reactive O_2_ species formed during the infection^50^. In addition, several natural flavonoids were reported to exhibit potent bactericidal potential against antibiotics resistant *H. pylori* strains^66^. Xanthones also are well documented to exhibit anti *H. pylori* activity^67,68^. Mangiferin xanthone reported in *I. confusa* PF^29^ is a very potent gastroprotective^69,70^ and anti inflammatory^71^ compound.

It is worthy to note that, the *H. pylori*, COX-1, COX-2, and NO inhibitory potentials of irigenin (P_3_), were much higher than that of PF from which it was isolated. Thus, fractionation and purification of *I. confusa* crude fractions caused significant increase in the aforementioned activities which may indicate a chance for isolation of more active constituents.

To the best of our knowledge, this is the first study that demonstrates the anti-*H. pylori* activity as well as COX-2 and IMPDH selective inhibitory activity of *I. confusa* underground parts and its isolated compounds.

Novel strategies are urgently required to control *H. pylori* infection. Our findings recommend further studies on *I. confusa* bioactive metabolites and also suggest irigenin as an important lead for management of *H. pylori* infection after detailed in vivo and clinical studies.

## Conclusion

Irigenin exhibited promising activity against *Hp*IMPDH enzyme with low activity against human *h*IMPDH2 than clarithromycin, assuring its selectivity in addition to it’s selective COX-2 inhibitory potential. This study scientifically validated the claimed anti-bacterial activity of *I. confusa* and has put strong focus on exploring traditional Chinese medicine for novel anti- *H. pylori* infections controlling drugs.

## Supplementary Information

Supplementary Information.

## Data Availability

All data generated or analysed during this study are included in this published article (and its Supplementary Information files).

## Abbreviations

COX-1 and COX-2: Cyclooxygenases
*H. pylori*: *Helicobacter pylori*
*h*IMPDH2: Human inosine-5′-monophosphate dehydrogenase
*Hp*IMPDH: *Helicobacter pylori* inosine-5′-monophosphate dehydrogenase
IMPDH: Inosine-5′-monophosphate dehydrogenase
MTT: 3-(4,5-Dimethyl-thiazol-2-yl)-2,5-diphenyl-tetrazolium bromide
NAD^+^: Nicotinamide adenine dinucleotide
NO: Nitric oxide
NPF: Non-polar fraction
PF: Polar fraction
WHO: World Health Organization
XMP: Xanthosine 5ʹ‐monophosphate
