# Supplementary material for: Irigenin, a novel lead from Iris confusa for management of Helicobacter pylori infection with selective COX-2 and HpIMPDH inhibitory potential
Source: Sci Rep. 2022 Jul 6;12:11457. doi: 10.1038/s41598-022-15361-w (PMC9259591; doi:10.1038/s41598-022-15361-w)
Supplement: Supplementary file 1 — Supplementary Information. [file 41598_2022_15361_MOESM1_ESM.docx]

# **Supplementary file**

# **Irigenin, a novel lead from *Iris* *confusa* for management of *Helicobacter pylori* infection with selective COX-2 and *Hp*IMPDH inhibitory potential**

Passent M. Abdel-Baki^1^, Moshera M. El-Sherei^1^, Amal E. Khaleel^1^, Marwa M. Abdel-Aziz^2^ and Mona M. Okba^1,^ *

*^1^ Department of Pharmacognosy, Faculty of Pharmacy, Cairo University, Cairo, 11562, Egypt.*

*^2^ Regional Center for Mycology and Biotechnology (RCMB), Al-Azhar University, Cairo, 11651, Egypt*

*Corresponding author: mona.morad@pharma.cu.edu.eg, mob No: +201002905055, fax No: +20225321900

**Material and methods**

**Determination of anti-*Helicobacter* *pylori* activity:**

The anti-*Helicobacter* *pylori* activity of the PF and NPF as well as the isolated compounds was evaluated against *H. pylori* ATCC 700392 (a type strain, obtained from the American Type Culture Collection unit (ATCC), using microwell dilution method and clarithromycin as standard drug ^1^. The tested samples were prepared in dimethyl sulfoxide and successive ten twofold dilutions (125- 0.24 µg/mL) were carried out in a 96-well plate. Each microplate well included forty μL of Brain Heart Infusion growth medium and 10% fetal bovine serum (FBS), ten μL of inoculum and fifty μL of the tested sample. The plates were incubated for 3 days, in 5% oxygen, 10% carbon dioxide, 85 % nitrogen atmosphere at 37°C. Forty μL of 3- (4, 5-dimethyl-thiazol-2-yl)- 2,5-diphenyl-tetrazolium bromide (MTT) at a final concentration of 0.5 mg/mL freshly prepared in H_2_O was added to each well and incubated for 30 min. The bacteria were biologically active when change to purple color was observed ^1^**.** While no change in MTT color indicated inhibition. Solvent control (DMSO) and the standard antibiotic (clarithromycin) were used as negative and positive controls, respectively. The *H.* *pylori* inhibition % was calculated using the given formula:

% Inhibition = Abs control – Abs sample × 100 /Abs control.

The absorbance (Abs) was observed at 620 nm. The concentration of samples required for 100% inhibition (MIC) values were done in triplicate.

**Determination of COX-1 and COX-2 inhibitory activity:**

The tested samples and the standard drugs (ibuprofen and celecoxib) were prepared in dimethyl sulfoxide and subsequent eight twofold dilutions (125- 0.98 µg) were carried out in a 96-well plate. The inhibitory COX activity was assayed colourimetrically by examining the inhibition of the ovine COX-1 and human recombinant COX-2 enzymes as described by George et al. ^2^**,** using a COX inhibitory screening assay kit. This assay determines the COX peroxidase activity by monitoring the appearance of N,N,N',N'-tetramethyl-*p*-phenylenediamine (TMPD)**.** Assay buffer **(**150 μL 0.1 M Tris–HCl pH 8.0), 10 μL heme and 10μL enzyme (COX-1 or COX-2) were added in each well. Ten μL of each tested sample concentration was added to each well and incubated for 15 minutes at 37°C. After incubation, 20 μL of TMPD was added to each well. For initiating the reaction, 10 μL 10 mM arachidonic acid was added and the plates were further incubated for another 2 minutes at 37°C. For terminating the reaction, 50 μL 1 N HCl and saturated stannous chloride were added.

Reaction mixture containing assay buffer and heme acted as the blank and a mixture with either COX-1 or COX-2 assay buffer and heme acted as the 100% initial activity (IA). Ibuprofen and celecoxib were run as the positive control for inhibition of COX-1 and COX-2, respectively. The absorbances of oxidized TMPD (blue colour) were read in ELISA plate reader at 590 nm. The % inhibition was calculated using the following formula:

COX % Inhibition= [(100%IA−Inhibitor)/ 100% IA x 100]. Where, IA: 100% initial activity.

The concentration at which the tested sample produces 50% inhibition of COX-1 and/or COX-2 (IC_50_) was determined. The selectivity index (SI) was calculated also as IC_50_ (COX-1)/ (COX-2).

**Determination of nitric oxide (NO) inhibitory activity:**

The macrophage cell line, RAW 264.7 was obtained from Vaccines, Sera and Drugs Egyptian Company (VACSERA). It was cultured in (RPMI, 1640) medium supplemented with 10% fetal bovine serum and 1% gentamicin. The cell was incubated at 37 °C in 5% carbon dioxide. RAW 264.7 cells were seeded onto a 96-well plate (1×105 cells/well) and incubated for 24 h. They were incubated for 1 h with the tested samples at concentrations of 125-0.24 µg/mL. Followed by incubation, the cells stimulated with 1µg/mL of lipopolysaccharide for another 24 h. The supernatant was gently transferred to new 96-well plates and used for NO determination. L-N 6 - (1-iminoethyl) lysine hydrochloride), is used as standard drug. The absorbance (Abs) was measured in a microplate reader at 540 nm ^3^. The percentage inhibition was calculated using the following formula:

Inhibition (%) = 1-[Control-Test] Control × 100.

The concentration at which 50% inhibition of NO (IC_50_) occurred was determined.

***In vitro Hp*IMPDH inhibition assay**

The most potent isolated compound, as revealed from the previous assays, was screened at different concentrations (10-0.078 µM) in triplicates according to Galal et. al. 2021^4^. The assay was carried out in a final volume of two hundred μL in black 96-well plate (Tarsons Products Pvt. Ltd., Kolkata, India) with a reaction buffer composed of one hundred mM KCl, fifty mM Tris–HCl (pH 8.6), and one mM dithiothreitol (DTT). One hundred nM *H. pylori* inosine-5′-monophosphate dehydrogenase (*Hp*IMPDH) in the presence/absence of the tested compound were used. The assay mixture was then incubated at 37 ºC for 10 min, and the reaction was initiated by the adding 250 μM of inosine 5ʹ‐monophosphate (IMP) and 300 μM of nicotinamide adenine dinucleotide (NAD^+^) substrate buffer. The assay was allowed to proceed for 45 min at 37 ºC.

The formed NADH was measured by recording the fluorescence (excitation 340 nm, emission 440 nm) using PerkinElmer EnVision Multilabel Reader (Waltham, MA) at an interval of one min. DMSO and clarithromycin were used as vehicle control and positive control, respectively. The concentration of the tested sample leading to 50% *Hp*IMPDH inhibition (IC_50_) was estimated.

***In vitro h*IMPDH2 inhibition assay**

Human inosine-5′-monophosphate dehydrogenase (*h*IMPDH2) was purchased from NovoCIB SAS (Lyon, France). It was used for the *in vitro* screening of the most potent isolated compound, as revealed from the previous assays, against *H. pylori* and the standard drug at the concentrations (10-0.078 µM) in triplicates ^4^. The assay was carried out in 96-well plates (Tarsons, 980040). The reaction buffer consisted of hundred mM KCl, hundred mM Tris–HCl (pH 8.6), and five mM DTT, 4% *v/v* DMSO with or without the tested compound and 0.15 mU of purified *h*IMPDH2 enzyme per well (from 1.5 mg/mL stock concentration). The enzyme stock solution final volume was 2 μL per well, which was insignificant to cause final assay buffer composition change. Addition of (substrate buffer) 0.2 mM of IMP and 0.2 mM of NAD^+^ initiated the assay, then it was allowed to proceed for 30 min at 37 ºC. The formed NADH was measured by reading the absorbance at 340 nm. At this λ, a background of <0.1 optical density was observed with the negligible cross-talk between wells. The concentration of the tested sample leading to 50% *h*IMPDH2 inhibition (IC_50_) was estimated.

| 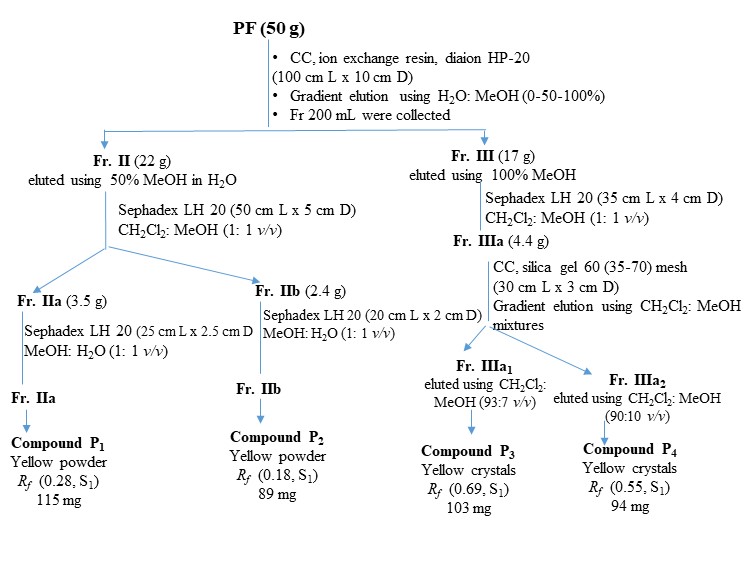 | 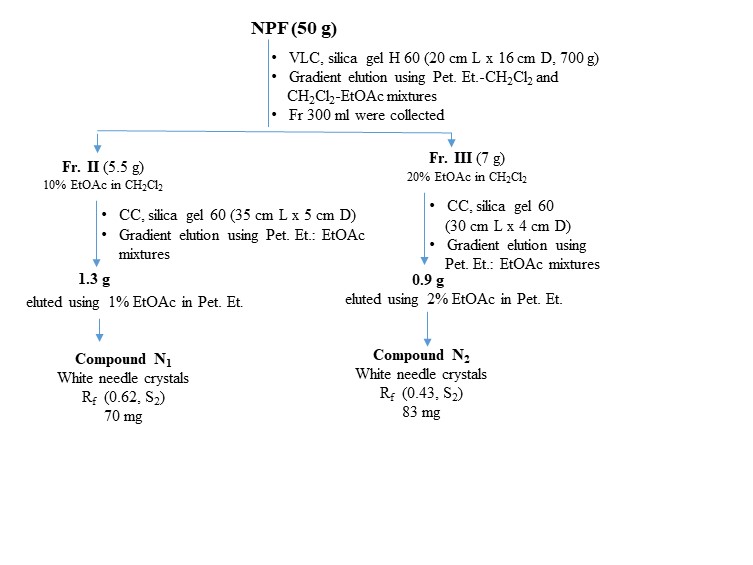 |
| --- | --- |

**Figure S1: Scheme for chromatographic fractionation of *I. confusa* underground parts polar fraction (PF) and non-polar fraction (NPF)** S_1_: methylene chloride-methanol-formic acid 85:15:0.2 *v/v/v*; S_2_: methylene chloride: methanol 97:3 *v/v*

**Results:**

**Table S1: Physicochemical, chromatographic and UV spectral data of compounds P_1_-P4**

|  | | **P1** | **P2** | **P3** | **P4** |
| --- | --- | --- | --- | --- | --- |
| **Physical properties** | **Condition** | Powder (115 mg) | Powder (89 mg) | Crystals (103 mg) | Crystals (94 mg) |
|  | **Colour** | Yellow | Yellow | Yellow | Yellow |
|  | **Melting range** | 256-259 °C | 265-268 °C | 190-191 °C | 225-226 °C |
|  | **Solubility** | Methanol | Methanol | Methanol | Methanol |
| **Colour reactions** | **UV (λmax 254)** | Deep purple | Deep purple | Deep purple | Deep purple |
|  | **UV/NH_3_** | Deep purple | Yellow-green | Deep purple | Deep purple |
|  | **AlCl_3_** | Yellow | Yellow | Yellow | Yellow |
| **R*_f_* values** | **S_1_** | 0.28 | 0.18 | 0.69 | 0.55 |
| **UV spectral data, λ_max_ (nm)** | **MeOH** | 267, 331 (sh) | 255,267,293(sh), 346 | 268, 336 (sh) | 267, 330 (sh) |
|  | **NaOMe** | 274, 365 | 268,278 (sh),334 (sh),405 | 273, 336 (sh) | 278, 328 (sh) |
|  | **AlCl_3_** | 277, 315(sh), 380 | 276, 302 (sh), 329, 429 | 275, 316(sh), 371 | 275, 320(sh), 374 |
|  | **AlCl_3_/HCl** | 278, 322(sh), 388 | 265 (sh), 276, 296sh, 357, 384 | 278, 315(sh), 374 | 277, 317(sh), 374 |
|  | **NaOAc** | 267, 331(sh) | 278, 325, 386 | 273, 338 | 271, 330 |
|  | **NaOAc/H_3_BO_3_** | 267, 329 (sh) | 264, 375, 430 (sh) | 268, 338 (sh) | 267, 324 (sh) |

P_1_: tectoridin (tectorigenin-7-*O*-*β*-D-glucoside); P_2_: orientin (luteolin-8-C-*β*-glucopyranoside); P_3_: irigenin (3′,5,7-trihydroxy-4′,5′,6 trimethoxyisoflavone); P_4_: tectorigenin (4′,5,7-trihydroxy-6-methoxyisoflavone); S_1_: methylene chloride-methanol-formic acid 85:15:0.2 *v/v/v*

**Table S2: ^1^H-NMR and ^13^C-NMR spectral data of compounds P_1-_P_4_**

|  | **P_1_** | | **P_2_** | | **P_3_** | | **P_4_** | |
| --- | --- | --- | --- | --- | --- | --- | --- | --- |
| **H/C** | **^1^H** | **^13^C** | **^1^H** | **^13^C** | **^1^H** | **^13^C** | **^1^H** | **^13^C** |
| **2** | 8.45 (s) | 155.17 |  | 164.16 | 8.40 (s) | 154.81 | 8.25 (s) | 153.76 |
| **3** |  | 122.54 | 6.64 (s) | 102.41 |  | 126.00 |  | 122.84 |
| **4** |  | 181.25 |  | 182.03 |  | 180.25 |  | 181.25 |
| **5** |  | 153.37 |  | 160.48 |  | 153.37 |  | 153.65 |
| **6** |  | 132.93 | 6.25 (s) | 98.33 |  | 131.44 |  | 131.52 |
| **7** |  | 157.10 |  | 162.80 |  | 157.50 |  | 157.53 |
| **8** | 6.90 (s) | 94.49 |  | 104.66 | 6.51 (s) | 93.94 | 6.45 (s) | 93.61 |
| **9** |  | 152.94 |  | 156.00 |  | 152.84 |  | 153.18 |
| **10** |  | 106.94 |  | 104.00 |  | 104.64 |  | 105.34 |
| **1'** |  | 121.51 |  | 121.97 |  | 121.65 |  | 121.51 |
| **2'** | 7.42 (d, *J*= 8.6 Hz) | 130.65 | 7.48 (d, *J*= 2 Hz) | 114.07 | 6.71 (d, *J*= 2 Hz) | 110.31 | 7.35 (d, *J*= 8.56 Hz) | 130.01 |
| **3'** | 6.85 (d, *J*= 8.6 Hz) | 115.56 |  | 145.95 |  | 150.18 | 6.82 (d, *J*= 8.6 Hz) | 114.97 |
| **4'** |  | 157.95 |  | 149.90 |  | 136.30 |  | 157.64 |
| **5'** | 6.85 (d, *J*= 8.6 Hz) | 115.56 | 6.85 (d, *J*= 8.6 Hz) | 115.78 |  | 152.62 | 6.82 (d, *J*= 8.6 Hz) | 114.97 |
| **6'** | 7.42 (d, *J*= 8.6 Hz) | 130.65 | 7.54 (dd, *J*= 2, 8.4 Hz) | 119.45 | 6.66 (d, *J*= 2 Hz) | 104.42 | 7.35 (d, *J*= 8.56 Hz) | 130.01 |
| **6-OCH_3_** | 3.78 (s) | 60.76 |  |  | 3.79 (s) | 60.31 | 3.75 (s) | 59.01 |
| **5-OH** | 12.94 |  | 13.24 (s) |  | 13.02 (s) |  | 13.06 (s) |  |
| **7-OH** |  |  |  |  | 10.91(s) |  | 10.75 (s) |  |
| **3'-OH** |  |  |  |  | 9.31(s) |  |  |  |
| **4'-OH** | 9.61 |  |  |  |  |  | 9.55 (s) |  |
| **4'-OCH_3_** |  |  |  |  | 3.75 (s) | 59.94 |  |  |
| **5'-OCH_3_** |  |  |  |  | 3.69 (s) | 55.74 |  |  |
| **1''** | 5.45 (d, *J*= 7.2 Hz) | 100.62 | 4.95 (d, *J*= 10 Hz) | 73.59 |  |  |  |  |
| **2''** | δ 3.7-3.1 | 73.60 | 4.07-3.51 (6H, m) | 70.90 |  |  |  |  |
| **3''** |  | 77.18 |  | 78.88 |  |  |  |  |
| **4''** |  | 70.12 |  | 70.83 |  |  |  |  |
| **5''** |  | 77.77 |  | 82.04 |  |  |  |  |
| **6''** |  | 61.13 |  | 61.77 |  |  |  |  |

^13^C (ppm) (100 MHz, DMSO-*d_6_*); ^1^H δ (multiplicity, *J*/Hz) (400 MHz, DMSO-*d_6_*); P_1_: tectoridin; P_2_: orientin; P_3_: irigenin; P_4_: tectorigenin

**Table S3: Physicochemical and chromatographic data of compounds N_1_-N2**

|  |  | **N_1_** | **N_2_** |
| --- | --- | --- | --- |
| **Physical properties** | **Condition** | Crystals (70 mg) | Crystals (83 mg) |
|  | **Colour** | White | White |
|  | **Melting point** | 296- 298 °C | 174- 176 °C |
|  | **Solubility** | Methylene chloride | Methylene chloride |
|  | **Liebermann-Burchard’s test** | Positive | Positive |
| **Colour reaction** | ***p*-Anisaldehyde/H_2_SO_4_/heat** | Violet | Violet |
| **R*_f_* value** | **S_2_** | 0.62 | 0.43 |

N_1:_ isoarborinol; N_2_^:^ stigmasterol; S_2_: methylene chloride: methanol 97:3 *v/v*

**Table S4: ^1^H-NMR and ^13^C-NMR spectral data of compounds N_1-_N2**

|  | **N_1_** | | **N_2_** | | |
| --- | --- | --- | --- | --- | --- |
| **H/C** | **^1^H** | **^13^C** | **^1^H** | **^13^C** |  |
| **1** |  | 39.40 |  | 37.26 |  |
| **2** |  | 27.45 |  | 31.90 |  |
| **3** | 3.08 (dd, *J*= 3.8, 10.5) | 79.01 | 3.49 (m) | 71.82 |  |
| **4** |  | 39.52 |  | 39.68 |  |
| **5** |  | 51.05 |  | 140.85 |  |
| **6** |  | 21.32 | 5.38 (d, *J*= 4.64) | 121.67 |  |
| **7** |  | 24.11 |  | 31.88 |  |
| **8** |  | 41.35 |  | 31.67 |  |
| **9** |  | 146.53 |  | 50.05 |  |
| **10** |  | 35.40 |  | 36.12 |  |
| **11** | 5.15 (d, *J*= 6.24) | 116.21 |  | 21.08 |  |
| **12** |  | 36.52 |  | 40.31 |  |
| **13** |  | 37.35 |  | 42.38 |  |
| **14** |  | 38.63 |  | 55.90 |  |
| **15** |  | 29.70 |  | 23.06 |  |
| **16** |  | 36.14 |  | 24.75 |  |
| **17** |  | 44.23 |  | 56.87 |  |
| **18** |  | 49.26 | 0.62 (s) | 11.98 |  |
| **19** |  | 20.50 | 0.98 (s) | 18.98 |  |
| **20** |  | 28.01 |  | 39.74 |  |
| **21** |  | 59.51 | 0.95 (d, *J*= 7.48) | 21.21 |  |
| **22** |  | 31.17 | 5.05 (dd, *J*= 8.52, 15.12 Hz) | 138.27 |  |
| **23** | 1.10 (s) | 28.04 | 4.94 (dd, *J*= 8.64, 15.16 Hz) | 129.33 |  |
| **24** | 0.77 (s) | 15.58 |  | 51.27 |  |
| **25** | 1.13 (s) | 22.83 |  | 29.70 |  |
| **26** | 0.74 (s) | 18.36 | 0.72 (d, *J*= 6.76) | 19.40 |  |
| **27** | 0.73 (s) | 15.33 | 0.76 (d, *J*= 8.48) | 19.81 |  |
| **28** | 0.72 (s) | 14.36 |  | 26.07 |  |
| **29** | 0.83 (d, *J*= 5.84) | 22.54 | 0.85 (t, *J*= 6.52) | 12.25 |  |
| **30** | 0.80 (d, *J*= 6.8) | 24.05 |  |  |  |

^13^C (ppm) (100 MHz, CDCl_3_); ^1^H δ (multiplicity, *J*/Hz) (400 MHz, CDCl_3_); N_1:_ isoarborinol; N_2_^:^ stigmasterol.

REFERENCE

1 Bonacorsi, C., Raddi, M. S. G., Carlos, I. Z., Sannomiya, M. & Vilegas, W. Anti-Helicobacter pylori activity and immunostimulatory effect of extracts from Byrsonima crassa Nied. (Malpighiaceae). *BMC Complement. Altern. Med.* **9**, 2, doi:10.1186/1472-6882-9-2 (2009).

2 George, A. *et al.* Anti-inflammatory effects of Polygonum minus (Huds) extract (Lineminus™) in in-vitro enzyme assays and carrageenan induced paw edema. *BMC Complement Altern Med* **14**, 355, doi:10.1186/1472-6882-14-355 (2014).

3 Chantaranothai, C., Palaga, T., Karnchanatat, A. & Sangvanich, P. Inhibition of nitric oxide production in the macrophage-like RAW 264.7 cell line by protein from the rhizomes of Zingiberaceae plants. *Prep. Biochem. Biotech.* **43**, 60-78 (2013).

4 Galal, A. M., Mohamed, H. S., Abdel‐Aziz, M. M. & Hanna, A. G. Development, synthesis, and biological evaluation of sulfonyl‐α‐L‐amino acids as potential anti‐Helicobacter pylori and IMPDH inhibitors. *Arch. Pharm.*, e2000385 (2021).
